# Advancing equity in allergy and immunology: progress, pitfalls, and the path forward

**DOI:** 10.3389/falgy.2025.1639718

**Published:** 2025-08-01

**Authors:** Philip Mendez, Ayobami Akenroye, Sharmilee M. Nyenhuis, Juan Carlos Cardet

**Affiliations:** ^1^ Division of Allergy and Immunology, Department of Pediatrics, University of South Florida Morsani College of Medicine, Tampa, FL, United States; ^2^ Division of Allergy and Immunology, University of South Florida at Johns Hopkins All Children’s Hospital, St. Petersburg, FL, United States; ^3^ Division of Allergy and Clinical Immunology, Brigham and Women’s Hospital, Boston, MA, United States; ^4^ Channing Division of Network Medicine, Brigham and Women’s Hospital, Boston, MA, United States; ^5^ Department of Medicine, Harvard Medical School, Boston, MA, United States; ^6^ Section of Allergy, Immunology, and Pediatric Pulmonology, Department of Medicine, University of Chicago, Chicago, IL, United States; ^7^ Division of Allergy and Immunology, Department of Internal Medicine, University of South Florida Morsani College of Medicine, Tampa, FL, United States

**Keywords:** health disparities, health equity, asthma, food allergy, atopic dermatitis, drug allergy, rhinitis, primary immunodeficiency

## Abstract

Health disparities in allergic and immunologic conditions are shaped by unequal exposure to social determinants of health (SDoH), including education, healthcare quality, neighborhood and built environment, social context, and economic stability. This review summarizes recent literature on disparities across asthma, food allergy, eosinophilic esophagitis, atopic dermatitis, allergic rhinitis, chronic rhinosinusitis, drug allergy, and primary immunodeficiency. Marginalized populations—including Black, Latinx, and low-income individuals—experience delayed diagnoses, limited access to specialist care, underuse of evidence-based therapies, and disproportionate exposure to environmental triggers. The manuscript highlights successful interventions including community health worker–led outreach, school-based programs, housing modifications, and policy reforms addressing affordability, housing, and environmental quality. However, recent cuts to federal agency staffing and funding jeopardize continued progress, threatening public health infrastructure that supports equitable care for many diseases. Sustained investment, interdisciplinary collaboration, and policy-driven strategies remain critical to addressing persistent inequities and improving outcomes in historically underserved communities.

## 1 Introduction

Health disparities are unjust and preventable differences in individuals' health status and outcomes (1). These disparities are largely driven by the unequal distribution of social determinants of health (SDoH) which include five key components: (1) Education Access and Quality, (2) Health Care and Quality, (3) Neighborhood and Built Environment, (4) Social and Community Context, and (5) Economic Stability. These components are deeply interconnected and operate within a broader, multi-level socio-ecological framework, ultimately increasing the risk of adverse outcomes in vulnerable populations (2). Although the role of SDoH in driving health disparities has been established for many allergic and immunologic conditions (3–6), these components are dynamic, evolving over time in their impact on patient health. Thus, it is essential for public health efforts to regularly evaluate and reassess which SDoH exposures most strongly influence health disparities in a given population, context, and time.

This article reviews the most recent evidence on health disparities in asthma, food allergy, eosinophilic esophagitis (EoE), allergic rhinitis (AR), chronic rhinosinusitis (CRS), atopic dermatitis (AD), drug allergy and primary immunodeficiency (PID). It also highlights emerging interventions aimed at addressing and mitigating these health disparities (Table 1).

**Table 1:** Summary of major U.S. policies and programs addressing disparities in asthma and food allergy.

Policy/program	Year implemented	Key provisions	Expected impact on asthma and food allergy outcomes
US EPA clean air act amendments	1990	Environmental policies to reduce pollution exposure	Reduce asthma exacerbations and the burden of allergic rhinitis and chronic rhinosinusitis
CDC's National Asthma Control Program (NACP)	1999 (Ongoing)	Currently it funds 29 programs. Uses the multi-pronged EXHALE framework to reduce ED visits and address disparities. This includes community- and school based interventions.	Improved education and adherence to asthma guidelines, reduced asthma-related missed work and school days.
EpiPen4Schools program	2012	Provides free epinephrine auto-injectors and training materials to schools. Over 50,000 autoinjectors distributed since inception.	Expanded access to emergency treatment of anaphylaxis
School access to emergency epinephrine act (H.R. 2094)	2013	Law encouraging states to have “stock” epinephrine in schools	Increased access to emergency treatment for allergic reactions. Lower adoption in underfunded school districts.
Environmental justice executive order (EJEO)	2021	Focused on uneven exposure to environmental hazards	Reduced exposure to pollution in all communities, including marginalized communities
School-based allergies and asthma management program act (H.R.2468)	2021	Supports comprehensive asthma education and care plans in schools	Reduced absenteeism and emergency visits for children with asthma
CHICAGO plan (community-based asthma intervention)	2017	Community-based program combining CHW follow-up for asthma patients with ED-based provider education.	Increased inhaler prescription refill rates, improved medication adherence
FASTER act (S.578)	2021	Mandated sesame allergen labeling, improved food allergy data collection	Better documentation of food allergy disparities, reduced allergic reactions
Food equality initiative	Ongoing	Provides free allergen-safe groceries to low-income families	Reduced food insecurity and improved nutrition for these families
Online peer-support networks (teen talks, allergy pals)	Ongoing	Virtual support groups for youth with asthma and allergies	Reduced social isolation, improved self-management strategies
Stock epinephrine laws (37 states)	Varies by state	Allows schools and public venues to stock epinephrine auto-injectors	Improved emergency response for anaphylaxis in children without personal devices
Expanded food assistance for allergen-free foods	Proposed/Ongoing	Advocates for reimbursement of specialized formulas and medical foods under insurance	Increased access to medically necessary allergen-free foods for families in need
Inhaler out-of-pocket cost caps, such as in Minnesota and Illinois	Varies by state	Caps the monthly cost of a single prescription inhaler at $25–35 for a 30-day supply	Increased affordability of asthma medication, reduced financial barriers

Federal, state, and community-based initiatives are listed with year of implementation, core provisions, and anticipated or observed health outcomes. These policies address disparities in asthma, food allergy, anaphylaxis, and immunologic care through strategies such as pollution control, CHW deployment, stock epinephrine legislation, and food assistance. CDC, centers for disease control and prevention; EPA, environmental protection agency; EJEO, environmental justice executive order; NACP, National Asthma Control Program; CHW, community health worker.

## 2 Asthma

### 2.1 Issues highlighted

**Figure 1:**
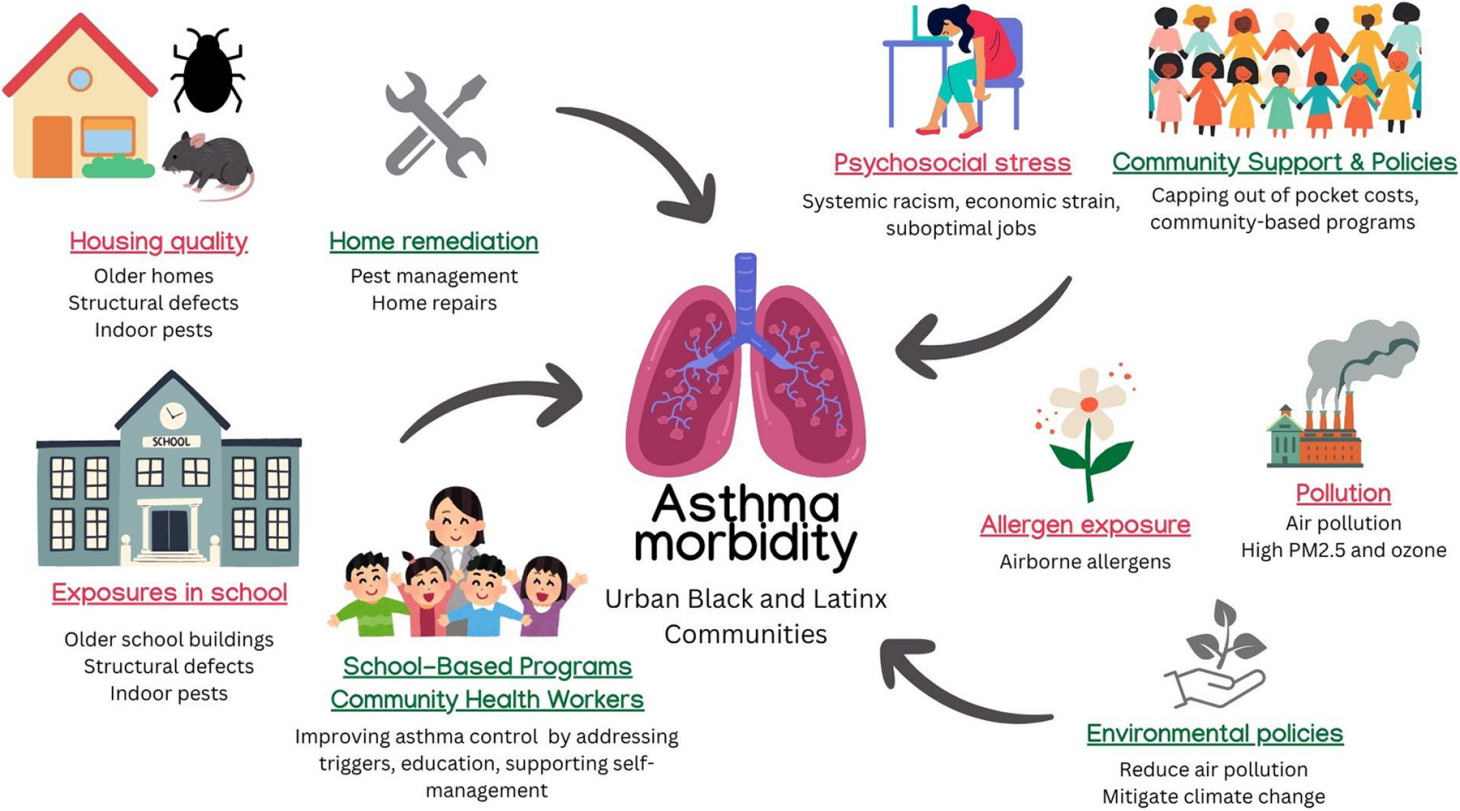
Effects of sDoH and interventions on asthma. Contributors to asthma morbidity in urban Black and Latinx communities include poor housing quality, school-based exposures, psychosocial stress, and air pollution. Policy and community-based interventions—such as environmental regulations, pest management, and school asthma programs—target these disparities through education, remediation, and access to care. ©[nomadion nomadd, irasutoya, iconsy, sketchify, Stelle Butalid, Isen Alejo, aunaun art studio, Jenzon Lopez, Camille Ramos, and RBTH] via http://Canva.com. PM2.5, particulate matter ≤2.5 microns; CHW, community health worker.

Limited health literacy is a persistent barrier to effective asthma control (Figure 1). Among adults with persistent asthma, poor health literacy has been associated with worse lung function, decreased quality of life, and higher emergency department (ED) utilization (7). Parents of Black children with asthma, particularly in urban communities, cite health beliefs regarding the safety and long-term side effects of asthma therapies as the most common barrier for management (8). Immigrant communities may also view medical management with skepticism due to language or cultural barriers. In a community-based participatory research study of Vietnamese American families, health-care professionals and community members reported diverging views on the benefits of Western therapy compared to traditional Vietnamese therapies and concerns about medication use, highlighting the need for more tailored, culturally competent educational strategies (9). Cultural practices around medication naming can also contribute to misunderstandings. One study found that nearly half of Black and Latinx adults used “non-standard” names for their inhalers, which can hinder effective communication with providers and increase the risk of medication misuse (10).

Disparities in healthcare access remain a central driver of inequities in asthma morbidity. Cultural and linguistic barriers within the healthcare system contribute significantly to asthma disparities among Latinx patients. A qualitative study of Hispanic caregivers of children with asthma along the Texas-Mexico border revealed that 60% of caregivers were unaware of what controller medications were. Many also reported difficulties distinguishing between controller and rescue inhalers and frequently voiced fears about long-term side effects such as medication dependency. These themes underscore the need for accessible, bilingual, and culturally responsive asthma education as a means to improve patient-clinician communication (11).

Even among Medicaid-enrolled children, high out-of-pocket costs, logistical constraints (e.g., transportation), and parental concerns over therapy side effects limit consistent controller medication use (8). Material hardships, such as food insecurity or housing quality, perpetuate higher allergen exposure and asthma severity among urban Black children (12). Furthermore, insurance coverage contributes to access to asthma-related therapies. Medicaid-enrolled children living in rural communities, especially Black children, have higher rates of ED visits for asthma exacerbations compared to their urban counterparts, as demonstrated in a study in North Carolina (13). A retrospective analysis from 2004 to 2016 revealed that patients in low-income areas spent a higher proportion of their income on emergency asthma care and less on controller medications, demonstrating cost-related underuse of preventive treatment (14).

Race-based adjustments in pulmonary function testing (PFTs) have sustained inaccuracies in diagnosis and treatment thresholds, contributing to poorer outcomes for non-White populations (15). By normalizing the reductions in lung metrics in non-White populations, these individuals may inadvertently be deemed as having normal lung function, which may delay diagnosis and obscure modifiable risk factors such as systemic racism (16). Environmental exposures contribute substantially to asthma morbidity in marginalized communities. Older homes, mobile homes, and high-rise apartments, including public housing structures, typically report more frequent pest infestations and have higher concentrations of pest allergens (17). Black, urban-dwelling children are more likely to reside in housing containing higher concentrations of indoor allergens, such as cockroach and mouse allergens, even after adjusting for health insurance, household income, and caregiver employment status (12). Poorly maintained housing (including mold or structural deficiencies) further drives asthma exacerbations in non-White individuals (18).

Disparities are further observed with outdoor environmental allergen and pollution exposure. Historically redlined neighborhoods, which are disproportionately inhabited by people of color, associate with higher rates of ED visits for asthma (19). Elevated levels of particulate matter (PM2.5) and ozone further compound morbidity, disproportionately impacting children of color in the context of climate change (20, 21).

Psychosocial factors and community stress can worsen asthma severity. High perceived stress, often stemming from systemic racism, economic strain, or suboptimal employment conditions, correlates with increased asthma severity and worse symptom control (22). In fact, high perceived stress has been found to mediate the association between lower socioeconomic status (SES) and higher asthma morbidity among Black and Latinx adults (22). Limited community support services, such as insufficient health education programs or lack of easily accessible community clinics, can amplify chronic stress and lead to inconsistent primary care follow-up.

### 2.2 Interventions and policies

Federal legislation such as the School-Based Allergies and Asthma Management Program Act (2021), promotes comprehensive asthma education and care plans in schools, aiming to reduce absenteeism and ED visits (23). Community and school-based investigational interventions, such as the School Inner-City Asthma Intervention Study (SICAS), focus on integrated pest management and use of high-efficiency particulate air (HEPA) filters (24). The limited success in improving symptom days shown in SICAS may have been due to lower-than-expected baseline allergen levels compared to other similar research interventions. Community health worker (CHW)–led interventions, as shown in a study by the West Philadelphia Asthma Care Collaborative, can significantly improve asthma medication adherence, symptom recognition, and disease control (25). Other school-based interventions that directly incorporate asthma self-management education for children have shown reductions in hospitalizations and ED visits (26). However, such interventions are limited by school policies that restrict asthma medication administration to nurses, delaying timely treatment.

The CDC's National Asthma Control Program (NACP) used the multi-strategy EXHALE framework to reduce ED visits and address health inequities. Expanding patient-centered medical homes, environmental policies, and CHW outreach constituted part of a broader public health push (27). EXHALE was adopted by 29 U.S. states, with policies and interventions implemented at the state and county levels. For example, in Kentucky, school nurse training on stock albuterol policies led to a 97% increase in school nurses' knowledge of asthma care and medication administration (28). In Utah, 58% of home visitation program participants with uncontrolled asthma achieved control within 12 months of the initial home visits, with ACT scores improved in 61%, and quality of life improved in all participants (29). In California, CHW-led education resulted in reduced hospital/ED visits (83%), improved asthma control (63%), and fewer missed days of work or school (70%) (30).

Several clinical trials have addressed disparities in asthma. For example, the PeRson EmPowered Asthma Relief (PREPARE) pragmatic study found that a Patient-Activated, Reliever-Triggered Inhaled CorticoSteroid (PARTICS) strategy of as-needed use of inhaled corticosteroids added to usual care reduced exacerbations in Black and Latinx adults with moderate-to-severe persistent asthma compared to usual care alone (31). School-based telemedicine (SB-TEAM) and video-based telehealth interventions have also improved symptom control and adherence in urban-dwelling Black and Latinx children with persistent asthma (32, 33).

Local policies facilitating free or reduced-cost home modifications (e.g., pest control services, mold mitigation) may directly decrease allergen exposure and improve outcomes. Integrated pest management (IPM) initiatives in low-income housing units in Baltimore and Boston that achieved ≥75% reduction in mouse allergen exposure were associated with a 1-year increase in prebronchodilator FEV₁ of 238 ml among mouse-sensitized children, compared to a 131 ml increase in households with <75% allergen reduction (34). Large-scale housing quality improvement projects, as demonstrated by a housing renovation program in Cincinnati, resulted in nearly a 50% reduction in childhood asthma prevalence in a neighborhood with previously substandard housing; asthma prevalence decreased from 12.7% to 5.9% after completing housing renovations (35).

Recent research has also highlighted disparities in access to inhalers and biologics based on insurance type. While some pharmaceutical companies have introduced a $35 monthly cap on out-of-pocket costs for asthma inhalers, these caps do not apply to publicly-insured Medicare or Medicaid patients, who are at the highest risk for severe asthma (36). However, even among those with full insurance coverage, cost remains a barrier: a 2025 national study found that 8.3% of fully insured adults reported difficulty affording asthma medications, with rates rising to 33% among those with partial or no insurance, underscoring that a $35 monthly cost may remain out of reach for many (37). Similarly, delays in biologic approval present additional barriers in timely care, disproportionately affecting those with public insurance or site-specific restrictions (38).

Stricter environmental regulations that reduce air pollutants such as PM2.5 and nitrogen dioxide may reduce asthma incidence and improve lung function. In the Southern California Children's Health Study, which tracked air pollution and asthma trends in nine communities from 1993 to 2014, reductions of 8.1 µg/m^3^ in PM2.5 and 4.3 ppb in nitrogen dioxide were significantly associated with 19% and 18% decreases in childhood asthma incidence, respectively. In five of these communities, a 14.1 ppb decrease in nitrogen dioxide was associated with a 91.4 ml increase in 4-year FEV₁ growth, while a 12.6 µg/m^3^ reduction in PM2.5 was associated with a 65.5 ml increase (39, 40).

Community-based programs such as the CHICAGO Plan, which combined ED-based provider education plus CHW follow-up, demonstrated significantly improved outcomes compared to usual care, achieving higher inhaler prescription fill rates (71% vs. 42%) and greater provider adherence to guideline-based management (40% vs. 10%) (41, 42). Culturally competent patient-provider communication training (e.g., cross-cultural communication methods) can also help improve providers' instructions about inhaler technique and addresses fears regarding inhaled corticosteroids (43). Policymakers can examine how to mitigate out-of-pocket costs for vulnerable patient populations. Medicaid health home models and community-based case management can mitigate treatment nonadherence related to cost and reduce preventable ED visits.

Beyond clinical care, community-level strategies such as housing support and affordability initiatives may offer alternative approaches to reducing asthma disparities. A public housing mobility program conducted in Baltimore relocated families from low-income to high-income neighborhoods and led to nearly a 50% reduction in asthma exacerbations and symptom days in children with asthma (44).

Several states have enacted legislation to cap out-of-pocket costs for asthma medications. Minnesota and Illinois have both enacted laws that cap out-of-pocket costs for prescribed asthma inhalers at $25 per 30-day supply. Minnesota's legislation went into effect on January 1st, 2025, while Illinois's will be effective in 2026 (45, 46). These laws aim to improve affordability for patients requiring maintenance and rescue therapies. However, a recent study from British Columbia found that eliminating out-of-pocket costs for asthma controller medications among low-income individuals did not significantly improve the proportion of days covered by controller therapy (0.01; 95% CI, −0.01 to 0.04) or meaningfully reduce excessive reliever use (−6.37%; 95% CI, −12.90 to 0.16), suggesting that factors associated with medication adherence are complex and cost reduction alone may be insufficient to improve long-term asthma control (47).

## 3 Food allergy and eosinophilic esophagitis

### 3.1 Issues highlighted

**Figure 2:**
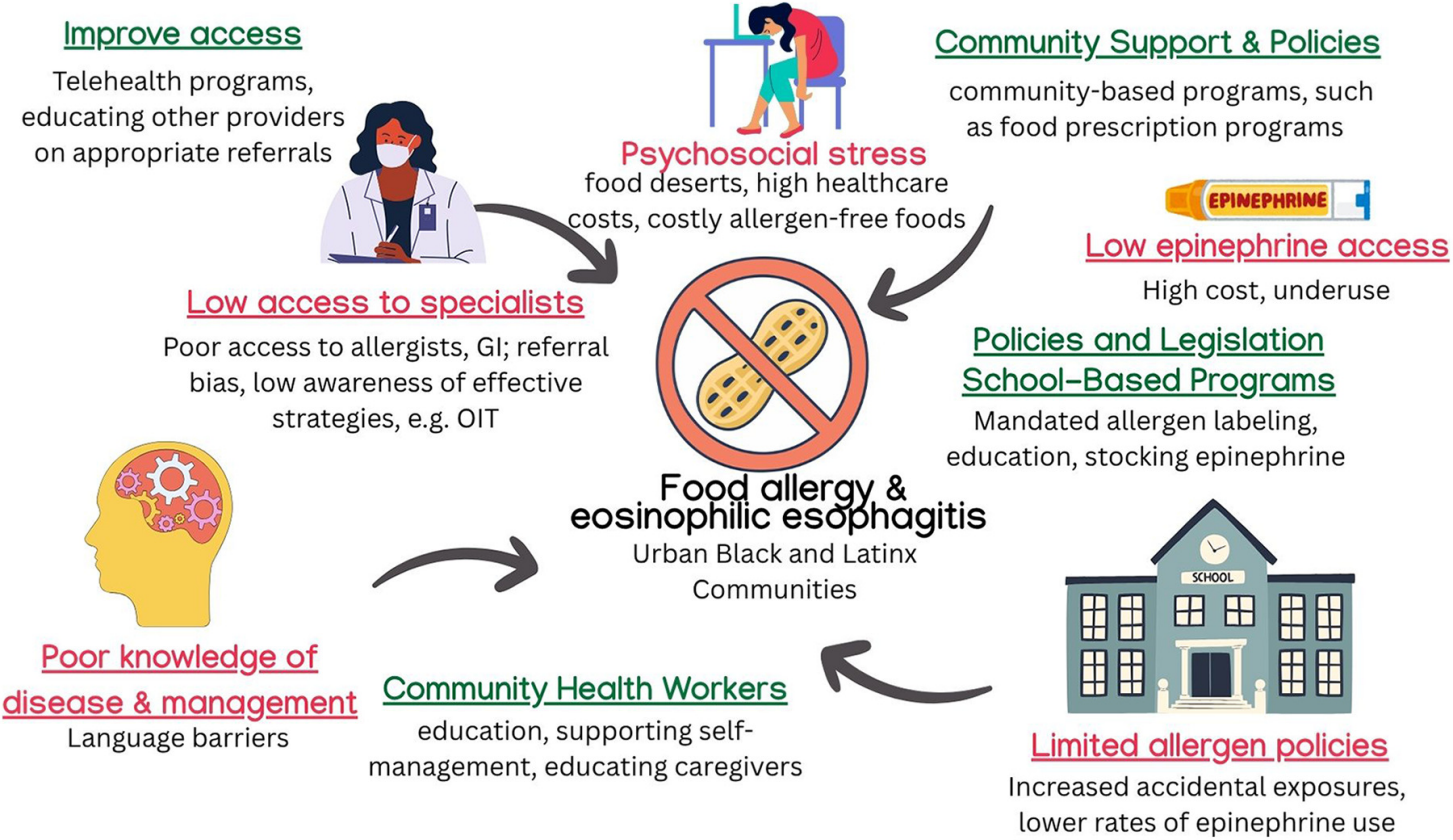
Effects of sDoH and interventions on food allergy. Barriers contributing to food allergy and EoE morbidity include limited specialist access, food insecurity, high epinephrine costs, and poor disease education. Interventions such as school-based supports, CHW outreach, telehealth, and allergen-safe food programs aim to reduce disparities and improve disease management in underserved populations. ©[sketchify, irasutoya, Camille Lopez, Harshit Chaudhry, Aira Borja, and Jenson Lopez] via http://Canva.com. OIT, oral immunotherapy; GI, gastroenterology; CHW, community health worker.

Families of patients with food allergies and EoE may lack adequate knowledge about early symptom recognition, safe dietary management, and epinephrine auto-injector use. Black and Latinx families, as well as those with limited English proficiency, encounter barriers to receiving timely allergy referrals and diagnostic workups (48). In many cases, families are unaware of specialized treatment options such as oral immunotherapy (OIT), and confusion around allergic symptom management can further contribute to underuse of epinephrine or delayed responses to allergic reactions (49). Socioeconomic disparities also play a role—lower-income and Black families face higher rates of ED visits and hospitalizations related to food allergy but are less likely to have access to personal epinephrine auto-injectors (50) (Figure 2).

Similar disparities are observed in EoE. Caregivers should understand available treatment strategies including swallowed corticosteroids, proton pump inhibitors, elimination diets, and biologic therapy. When health literacy is limited, families may be less likely to pursue follow-up endoscopy or feeding therapies, especially when these services are not clearly explained, culturally tailored, or financially accessible (51).

Non-White children living in low-income, urban neighborhoods are less likely to undergo formal allergy testing or receive evaluation by an allergist. In a New York City study, 3.4% of children had a physician-documented food allergy, with Black children exhibiting a 1.5-fold higher prevalence compared to Latinx, Asian, and White children; however, fewer than half of the children received confirmatory testing or allergist evaluation (48). Similarly, a survey of over 38,000 children found that Black and Asian children were significantly more likely to have parent-reported food allergy than White children, with adjusted odds ratios (aOR) of 1.8 and 1.4, respectively. Yet among children with reported food allergy, those who were Black (aOR 0.8), Asian (aOR 0.7), or Hispanic (aOR 0.8) had lower odds of having a confirmed diagnosis, defined by physician evaluation and/or allergy testing, compared to White children. Children from households earning less than $50,000 annually also had half the odds of receiving a confirmed diagnosis compared to those from higher-income households (aOR 0.5) (49).

Black and Latinx children are less likely to receive OIT or undergo oral food challenges (OFC), indicating possible referral biases or lack of access to allergists. In one institutional registry of over 25,000 food allergy patients, those undergoing OIT and OFC were predominantly White (84.5% and 87.1%, respectively), with lower representation of Black (5.5% OIT; 7.5% OFC) and Asian (8.2% OIT; 5.4% OFC) patients (52). In a separate study, White patients were overrepresented in the OIT group compared to controls (75% vs. 63.6%), while Black (4.1% vs. 18%) and Asian (5.8% vs. 9.6%) patients were underrepresented (53).

For EoE, children from impoverished or rural communities often receive fewer endoscopic evaluations and are more likely to experience delayed diagnosis (54, 55).

Schools play a critical role in food allergy safety, yet inequitable access to resources persists. White children are nearly five times more likely to be dispensed epinephrine for peanut or tree nut allergy compared to non-White children (56). In a large urban district, schools serving higher-income students were six times more likely to have epinephrine autoinjectors available, even after adjusting for food allergy prevalence (57). In Massachusetts, Bartnikas et al. found that peanut-free tables were implemented in 91% of surveyed schools and were more common in schools with higher proportions of low-income (median 31.7% vs. 16.8%) and minority students (24.6% vs. 11.1%) (58). The authors cite these targeted policies as associated with lower rates of epinephrine administration for peanut and tree nut reactions. However, broader bans on peanuts showed no such association, highlighting challenges in enforcement. Importantly, the authors caution that lower epinephrine use may not reflect fewer reactions but rather systemic limitations, such as reduced epinephrine availability, staffing shortages, and financial constraints. These findings underscore how schools serving vulnerable populations may adopt restrictive policies in response to resource scarcity, raising important questions about equity and safety in food allergy management (Figure 2).

Households of patients with food allergies in underserved communities struggle to obtain allergen-free foods, particularly when relying on public benefits that do not cover these specialty items. This gap in coverage increases the risk of accidental exposures and contributes to healthcare costs that are approximately 2.5 times higher than those incurred by middle- and high-income households (50). Additionally, food deserts create further challenges for families managing food allergies or elimination diets. The U.S. Department of Agriculture (USDA) defines a food desert as a low-income census tract where at least 500 people or 33% of the population live more than one mile (in urban areas) or ten miles (in rural areas) from the nearest supermarket or large grocery store (59). Most recent data from the USDA estimates that 6.1% of the U.S. population, or 18.8 million people, reside in food deserts. While some studies suggest that food deserts do not directly correlate with food allergy outcomes, food insecurity significantly heightens psychosocial stress and food allergy-related anxiety (60, 61).

Food insecurity is a major concern among food-allergic households, with prevalence rates nearing 70% in some study cohorts (61). Cost barriers make allergen-free foods prohibitively expensive for many families. Despite the availability of school meal programs, many eligible families opt out due to fears of accidental allergen exposure. A survey found that 70% of eligible families declined participation in the National School Lunch Program (NSLP) or School Breakfast Program (SBP), citing concerns about food allergies as the primary reason (62). This is particularly concerning given that approximately 14.3 million and 19.7 million U.S. children benefit from the SBP and NSLP, respectively, with low-income children representing 60%–70% of participants (63, 64).

For children with EoE, geographic disparities further limit access to necessary care. Families living in areas with few pediatric gastroenterology clinics may have to travel long distances for repeated endoscopies or feeding therapy visits, posing logistical and financial barriers (51). In rural or high-area deprivation index (ADI) regions, lack of specialist access can delay critical procedures such as esophageal dilation, worsening long-term outcomes.

Programs such as EpiPen4Schools, which has provided free epinephrine auto-injectors and training materials to over 50,000 schools since 2012, have expanded access to emergency treatment. However, data on the racial, ethnic, and socioeconomic demographics of participating schools remain unavailable, leaving gaps in understanding the impact of this program on the most vulnerable communities (65).

### 3.2 Interventions and policies

The School Access to Emergency Epinephrine Act (2013) provided a legal framework for stocking epinephrine auto-injectors in schools (66). However, implementation has been inconsistent, with lower adoption rates in underfunded school districts, particularly those without full-time nurses (67, 68). Strengthening school-based anaphylaxis training and standardized epinephrine stocking policies could reduce disparities in emergency response during allergic reactions.

For EoE, standardizing educational outreach on allergic triggers, swallowed corticosteroids, and the importance of endoscopic surveillance may improve early intervention and reduce complications. While structured caregiver education programs for EoE remain limited, models from AD caregiver education programs suggest that structured training programs can improve disease management in low-literacy communities (69).

The Food Allergy Safety, Treatment, Education, and Research (FASTER) Act of 2021 mandated sesame allergen labeling and strengthened data collection on food allergy disparities (70). Additionally, nonprofit organizations such as the Food Equality Initiative have piloted “food prescription” programs, providing free allergen-safe groceries to reduce food insecurity for low-income families (71).

Stock epinephrine laws now exist in 37 states, allowing schools and public venues (e.g., recreation centers, summer camps) to carry epinephrine auto-injectors. Given that nearly half of school-based anaphylactic events involve students without a personal device, expanding these policies—particularly in schools serving minority and low-income populations—could improve outcomes (72).

Telehealth programs connecting families with pediatric gastroenterologists for EoE management can help mitigate geographic disparities. Virtual consultations reduce the need for long travel distances and improve adherence to long-term dietary and pharmacologic therapy (51).

Addressing food insecurity and allergen-free grocery access is critical. Mobile food markets, grocery cooperatives, and local community partnerships can help families in food deserts access safe dietary options. Some pilot programs offer “tailored grocery prescriptions” in collaboration with food pantries, further expanding options for food-allergic families.

Online peer-support networks, such as Teen Talks and Allergy Pals, have demonstrated success in reducing isolation and providing education in a supportive environment (73, 74). Linking these programs to local community centers could expand access for children in underserved areas. Additionally, standardized school-wide allergen labeling, designated allergen-free lunch tables, and mandatory staff training on allergy management help create safer and more inclusive environments for food-allergic students.

Expanding food assistance programs to accommodate allergen-free foods would ensure that low-income families can safely participate in federal meal programs. Advocacy efforts continue to push for expanded reimbursement of specialized formulas and medical foods for EoE under both private and public insurance plans (54). Addressing these financial barriers is crucial for improving long-term outcomes and reducing disparities in food allergy and EoE management.

## 4 Atopic dermatitis

### 4.1 Issues highlighted

Misdiagnosis of AD in patients with darker skin tones remains a pressing issue, often stemming from insufficient training in medical education on diverse dermatologic presentations. Dermatology residents consistently report lower confidence in diagnosing and managing skin conditions in patients with darker skin tones compared to doing so in White patients (75). In the same study, the authors found that structured education, such as dedicated didactics or clinical rotations, was associated with significantly higher resident confidence in diagnosis, yet fewer than one-third of programs offered such educational opportunities. This diagnostic bias can result in delayed or inappropriate care for Black, Latinx, and Indigenous patients (76). Furthermore, educational disparities among caregivers, particularly in Latinx households where parents were 79% less likely to have a bachelor's degree or higher compared to white households, may contribute to limited understanding of AD management, reducing treatment adherence and increasing disease burden (77).

Black and Hispanic children with AD are 3.4 and 1.5 times more likely, respectively, to have school absenteeism attributable to atopic dermatitis relative to non-Hispanic White children, even after adjusting for sociodemographic variables, healthcare utilization, disease severity, and atopic comorbidities (78). Compared to commercially-insured children, Medicaid-insured children with AD are over five times less likely to see a dermatologist for diagnosis (3.2% vs. 18.7%) and are more than three times as likely to have AD-related emergency department visits (10.8% vs. 3.2%) and be prescribed systemic antihistamines (70.9% vs. 21.9%). They were also significantly less likely to receive high-potency topical corticosteroids (12.9% vs. 16.5%) and calcineurin inhibitors (3.3% vs. 7.4%), which may indicate disparities in appropriate, recommended treatments (79). In terms of advanced therapies, Black patients with AD are more than twice as likely to require initiation of dupilumab therapy compared to White patients, reflecting higher disease burden (80).

Outdoor and indoor mold exposures can strongly influence AD development and severity. As demonstrated in a birth cohort study, prenatal mold exposure increased the odds of developing AD in infancy by 36% (adjusted OR 1.36; 95% CI 1.01–1.83), with exposed infants exhibiting significantly higher total serum IgE levels at 1 year of age (124.96 ± 413.82 kU/L) compared to unexposed healthy infants (58.71 ± 126.25 kU/L), suggesting an IgE-mediated mechanism of allergic inflammation (81). These risks are especially pronounced in lower-income households, which are more likely to reside in older, poorly ventilated homes located in flood-prone areas, where mold proliferation is common and post-flood remediation is often inadequate (82).

Outdoor air pollution and climate-related environmental disparities further contribute to AD risk. A national exposure model found that Black, Hispanic, and Asian populations are exposed to 21%, 11%, and 18% more PM2.5, respectively, than the U.S. average, while White populations are exposed to 8% less; these disparities persist across income levels and pollution sources (83). Children living in ZIP codes with industrial emissions one standard deviation above the national mean had a 7.7-fold higher rate of pediatrician visits for AD, and those residing within 50 m of a major roadway had a 4-fold increased risk of AD (84) These risks are not evenly distributed: nearly 40% of non-Hispanic Black children live in high-traffic neighborhoods and attend schools within 100 m of major roadways, heightening their exposure to allergenic and irritant pollutants (85). Additionally, low-income urban neighborhoods have on average 15.2% less tree cover and are 1.5°C hotter, with disparities reaching 30% less canopy and 4.0°C higher temperatures in northeastern cities (86).

It is important to also consider the role of neighborhood stressors and structural neglect with AD. In a nationally representative study of over 79,000 U.S. children, those living in neighborhoods with vandalism (aOR 1.28), litter (1.18), or poorly maintained housing (1.14) were significantly more likely to have AD (87). Children lacking neighborhood social cohesion, such as adults to trust or neighbors who help each other, had elevated odds of AD (aORs 1.16–1.32). These associations were magnified in children from single-parent or step-parent households, where the combination of family structure and environmental neglect resulted in up to a 2.7-fold increased risk of AD. Biologics for AD remain underutilized among publicly insured and non-White patients, despite these populations experiencing a higher disease burden (88).

### 4.2 Interventions and policies

School-based educational programs targeting AD management for both children and caregivers have demonstrated improved disease control, treatment adherence, and quality of life. These interventions emphasize medication use, skincare routines, and trigger avoidance (69). Online behavioral tools like the “Eczema Care Online” platform have also shown sustained benefits in eczema severity, self-management confidence, and POEM scores, particularly when tailored to children or young adults—with improvements maintained up to 52 weeks (89). WhatsApp-based digital interventions among Hispanic families led to a 14% improvement in AD knowledge, although retention declined after 1 month—presenting both the promises and challenges of digital health education (90).

Culturally-tailored care through community health workers, such as “promotoras de salud”, has proven highly effective in improving AD management in Spanish-speaking Latinx children. Interventions that included emollient use, wet wrap therapy, and bleach baths increased adherence and reduced disease burden (91, 92). Telemedicine initiatives have also demonstrated parity with in-person visits in terms of disease outcomes while offering additional benefits such as improved accessibility, reduced wait times, and higher patient satisfaction—making them a key tool in expanding equitable dermatologic care (93).

## 5 Allergic rhinitis, chronic rhinosinusitis, drug allergy and primary immunodeficiency

### 5.1 Issues highlighted

Poor health literacy is a well-documented barrier to effective disease management and is strongly associated with lower SES in allergic and immunologic diseases (94). Limited knowledge about guideline-recommended therapies results in suboptimal treatment adherence and worsened disease outcomes. Among low-income, minority children with persistent asthma, 63% also had allergic rhinitis (AR), yet only 44% received any medication for AR. Of those treated, the majority (68%) were given second-generation oral antihistamines, while just 28% received first-line therapy with intranasal corticosteroids (95). Disparities in allergen immunotherapy initiation also persist: Black and Latinx adults with AR are 60% and 20% less likely, respectively, than non-Hispanic White adults to start subcutaneous immunotherapy (SCIT) (96). Even among those who receive SCIT, Medicaid recipients miss an average of 34.2% of injections, nearly 10% more than Medicare patients (24.4%) and about 15% more than commercially insured patients (19.9%), with payor status significantly predictive of adherence rates (97). Access to care plays a vital role in CRS disparities. A study from Chicago found that African American patients had less frequent follow-up visits and worse SNOT-22 scores after 40 months of care compared to White patients (98). Similarly, Black and Medicaid-insured patients with CRS demonstrated more severe disease histopathologically and worse symptom scores, though these disparities were no longer statistically significant after adjusting for insurance status, underscoring the role of healthcare access in CRS morbidity (99). Hispanic patients in South Florida presenting for endoscopic sinus surgery reported longer delays in receiving care, greater CRS severity, and overall higher disease burden compared to non-Hispanic patients (100). Air pollution, which disproportionately affects low-income and minority communities, has also been linked to worsened CRS severity and higher oral steroid use for symptom control (101). In the Southeast U.S., Black patients with allergic fungal rhinosinusitis (AFRS) were more likely to be uninsured or rely on public insurance compared to non-Black CRS patients, reflecting structural barriers to timely surgical and specialty care (102).

Disparities stemming from prenatal and maternal exposures, such as material hardship and household smoke, shape the earliest trajectories of pediatric rhinitis. In a birth cohort from low-income New York City neighborhoods, children with persistent or late onset–frequent rhinitis were more likely to experience prenatal material hardship (57%) and allergic sensitization (63%) (103). These groups, which disproportionately included African American patients, had markedly elevated asthma risk: over 90% with persistent rhinitis and nearly 75% with late onset–frequent rhinitis were diagnosed with asthma. Compared to those with infrequent rhinitis, children with persistent symptoms had 64 times the odds of frequent wheeze, and among atopic children, 225 times the odds of physician-diagnosed asthma. These associations remained significant even after adjusting for maternal asthma, prenatal smoke exposure, and material hardship, highlighting that rhinitis trajectory independently contributes to asthma risk beyond shared environmental and genetic factors. Moreover, mouse sensitization has been linked to worsening rhinitis symptoms in urban-dwelling children with poorly controlled asthma, further demonstrating the intersection of environmental risk factors and allergic disease morbidity (104).

Barriers to penicillin allergy evaluation and delabeling are also shaped by SDoH. A retrospective cohort study of hospitalized children found that Black children and those with a non-English language preference had lower odds of carrying a penicillin allergy label compared to White and English-speaking children (105). While this observation may suggest different rates in penicillin allergies, it more likely reflects underdiagnosis and disparities in healthcare utilization and communication. Further, efforts to expand penicillin de-labeling into outpatient and primary care settings face ongoing challenges due to concerns over cost, provider time, and liability often limiting implementation, particularly in safety-net systems (106). Although inpatient de-labeling initiatives have shown success, broader adoption remains limited. In response, the PAVE Act (107) was introduced to incorporate penicillin allergy verification into initial Medicare preventive visits, though its implementation challenges remain uncertain.

Disparities in primary immunodeficiency (PID) diagnoses and treatment further underscore inequities in healthcare access. At a safety-net hospital, only 30.1% of patients with primary antibody deficiency (PAD) received immunoglobulin replacement therapy (IgRT), compared to 86.8% of patients in the USIDNET registry (108). Even among those with severe disease (IgG < 500), IgRT usage at the safety-net hospital was markedly lower (39.3% vs. 94.1%). Black patients with PAD were disproportionately affected, with 75% experiencing pneumonia and 75% developing bronchiectasis—rates more than fivefold higher than among other patients in the same cohort and far exceeding national registry benchmarks. These findings highlight how systemic barriers in under-resourced settings contribute to preventable complications and poorer outcomes among historically marginalized populations. Additionally, patients in non-White and high ADI neighborhoods experienced longer wait times for IgRT and had more severe PID-related complications, such as sepsis and pneumonia (109).

### 5.2 Interventions and policies

Addressing disparities in allergic and immunologic diseases requires a multifaceted approach that integrates community-based education, technological innovations, legislative reforms, and environmental policies. Expanding culturally tailored CHW programs has shown significant promise in improving adherence to AD treatments, such as wet-wrap therapy and emollient use, among Spanish-speaking families (91, 92). Similar initiatives could be implemented for AR, CRS, and drug allergy de-labeling to ensure that non-English-speaking patients receive accessible, high-quality care. Digital and telemedicine-based interventions may further enhance disease management; structured educational programs and mobile health interventions have been shown to improve INCS adherence in AR, though their impact on symptom control remains inconsistent (110, 111). In AD, telemedicine consultations have been demonstrated to provide comparable outcomes to in-person visits while improving access, reducing wait times, and increasing patient satisfaction (93).

The use of artificial intelligence (AI) in early screening for PIDs presents another promising avenue for reducing diagnostic delays. Recent studies have demonstrated that machine learning and natural language processing algorithms can identify patients with inborn errors of immunity up to 36 months earlier than traditional diagnostic methods (112–114). Incorporating AI-based screening tools into primary care and safety-net hospitals could facilitate earlier specialist referrals and reduce severe complications especially among non-White patients, who have historically experienced diagnostic delays in PID (109).

Legislative efforts play a crucial role in expanding access to effective treatments. The PAVE Act (107), introduced in 2024, proposes expanding penicillin allergy verification as part of Medicare's initial preventive physical exam visit. This initiative could significantly reduce unnecessary antibiotic restrictions, particularly among publicly insured patients who face higher barriers to penicillin allergy de-labeling. However, widespread implementation remains limited by provider concerns about cost, time, and liability in outpatient settings, particularly in safety-net primary care clinics where these services would be most beneficial (106). Additionally, H.R.2617 introduced a permanent bundled Medicare payment model that covers IgRT, associated supplies, and nursing services for home-based administration (115). Expanding this model to Medicaid and commercial insurers would alleviate financial burdens for patients with PIDs, ensuring more equitable access to life-saving therapies (108).

Environmental interventions are critical for mitigating exposure to allergens and pollutants that disproportionately affect lower-income and racially minoritized communities. Enforcing air quality regulations to reduce PM2.5 and other pollutants has the potential to lower the burden of AR, CRS, and asthma. A recent analysis projects that healthcare utilization for AR will increase by 41,000 visits annually if global warming reaches 2°C, disproportionately affecting limited English-speaking, Black, Indigenous, and People of Color (BIPOC), and uninsured children exposed to oak pollen (21). The use of indoor air purifiers has been shown to improve nasal symptoms in children with allergic rhinitis by reducing PM2.5 concentrations (116). Additionally, increasing public funding for housing remediation in flood-prone coastal regions could mitigate mold exposure, which has been associated with heightened AD risk, particularly among low-income families (81, 82, 86).

## 6 Pharmacoequity

Achieving justice in allergic and immunologic care requires pharmacoequity—the principle that all individuals, regardless of race, ethnicity, or socioeconomic status, should have access to the highest-quality medications needed to manage their health (117). Yet in practice, deep inequities persist, particularly in the treatment of allergic diseases and primary immunodeficiency. Despite having higher disease burden, publicly insured and non-White patients are less likely to be prescribed biologics for moderate-to-severe asthma and AD (88). The delayed approval process for biologics further exacerbates disparities, disproportionately affecting marginalized communities due to site-specific policies and insurance restrictions (38). Cost-saving models suggest that an over-the-counter option for budesonide-formoterol for mild asthma could generate $70.29 billion in lifetime healthcare savings, yet pharmaceutical companies' $35 monthly out-of-pocket cap for certain inhalers excludes Medicaid beneficiaries, placing the highest financial burden on those least able to afford care (36, 118). Addressing these inequities will require policy-driven efforts to reform insurance coverage criteria, streamline prior authorization processes, and ensure that Medicaid patients are included in cost-reduction programs for asthma and biologic therapies.

## 7 Discussion and conclusions

Morbidity from allergic diseases and primary immunodeficiency are profoundly influenced by SDoH. As outlined previously, key barriers such as limited education, inadequate health care access, substandard housing, social marginalization, and economic instability contribute to the disproportionate burden of disease in marginalized communities. Despite the implementation of evidence-based interventions—including CHW programs, telemedicine, environmental remediation, and policy reforms—recent cuts in federal staffing and funding threaten to undermine progress and widen existing disparities.

One of the most concerning developments in 2025 is the reduction in federal support for agencies such as the CDC, NIH and EPA, and potential additional reductions to Medicare and Medicaid. Historically, the NACP has helped implement multilevel strategies, including school-based interventions and CHW-led outreach, to reduce asthma morbidity and address healthcare inequities. With fewer resources and staff, these programs will struggle to maintain data collection, disseminate best practices, and coordinate community-level interventions, potentially reducing CHW-led education efforts and limiting environmental trigger mitigation strategies (25, 35).

Similarly, the EPA's efforts to mitigate climate change and enforce air quality standards are crucial for conditions such as asthma, allergic rhinitis, and atopic dermatitis, given the well-documented role of air pollution and environmental injustice in exacerbating allergic disease morbidity. Historically redlined neighborhoods, predominantly home to Black and Latinx populations, have been disproportionately affected by poor air quality, increased allergen exposure, and climate-related health risks (20). If federal staffing shortages impede pollution monitoring and enforcement, these exposures will likely worsen, exacerbating allergic and respiratory disease morbidity in under-resourced communities. Large-scale housing initiatives, such as IPM programs in public housing, also rely on interagency collaboration, and cuts in federal support could limit these interventions, leaving low-income households without adequate resources to reduce allergen exposure (19, 82).

In clinical care, reductions in NIH funding will have a profound impact on research funding for novel therapeutics and equitable clinical trial design. Black and Latinx patients remain underrepresented in clinical trials for emerging biologics for asthma, chronic rhinosinusitis, atopic dermatitis, and food allergy (119, 120). A decline in federal support could stall ongoing efforts to expand trial recruitment and assess real-world efficacy in historically marginalized populations. Furthermore, funding constraints could hinder capacity-building initiatives aimed at increasing the number of allergy/immunology specialists trained in culturally competent care, which is especially crucial in rural and medically underserved communities.

These disparities may also be shaped by genetic and epigenetic differences, including stress-induced modifications to immune regulatory pathways that are more prevalent in communities facing chronic adversity. Although the evidence base is still developing, incorporating genetic and epigenetic data into disparities research may help identify biologic contributors to unequal disease burden. Expanding representation in genomic studies is critical to ensuring that emerging diagnostics and therapeutics are equitable and applicable across diverse populations.

Staff reductions in federal agencies threaten the infrastructure necessary to support equitable public health communication and allergy care delivery. These cuts may hinder efforts to close long-standing knowledge gaps in drug allergy labeling, particularly within historically marginalized communities. Programs that rely on coordinated outreach, such as CHW-led efforts or telehealth initiatives, require sustained federal investment to function effectively. Without adequate staffing and funding, progress toward implementing accessible, community-based drug allergy evaluation services risks stalling, potentially reinforcing existing disparities in antimicrobial stewardship and allergy care.

Economic stability is another critical domain where reductions in federal investment could worsen disparities. For example, biologic therapies for asthma and atopic dermatitis remain less accessible to publicly insured and non-White patients, despite the higher disease burden in these populations (88). Policy measures that ensure IgRT coverage for patients with PIDs, or that guarantee access to biologics for severe allergic disease, require consistent funding and oversight. If federal staffing reductions affect insurance regulation, drug price negotiations, and prior authorization processes, patients in publicly funded programs like Medicaid may face delays or outright denials of coverage, leading to increased disease severity and preventable hospitalizations (80, 115).

While this review centers on literature from the United States, future research would benefit from international comparisons of allergic and immunologic disease disparities. Examining outcomes in countries with universal healthcare systems could help assess whether such disparities persist, even when access to care is more equitable. These comparisons may offer insight into the impact of broader societal, structural, or environmental factors that continue to influence health outcomes despite more uniform healthcare access.

The federal programs and agencies that combat health disparities in allergic disease and primary immunodeficiency represent a delicate ecosystem that could rapidly unravel without sufficient resources. Staffing shortages do not just reduce workforce capacity—they constrain the ability of agencies to implement programs in culturally tailored ways, further marginalizing vulnerable populations. In turn, inequities in the diagnosis and management of asthma, food allergy, atopic dermatitis, drug allergy, and PID could deepen, placing low-income, racially minoritized communities at disproportionate risk of disease progression, high healthcare costs, and preventable morbidity.

Looking forward, sustained investment in public health infrastructure is critical to mitigating these disparities. Maintaining robust funding and staffing levels across agencies such as the CDC, EPA, and NIH is essential for ensuring that evidence-based interventions, SDoH-focused policies, and equitable healthcare strategies remain in place. Without these measures, the considerable progress made in reducing allergic disease burden over the past decades is at risk of stagnation, ultimately reinforcing long-standing health inequities in allergy and immunology. Ensuring continued federal investment, interdisciplinary collaboration, and policy-driven reform will be necessary to prevent further deterioration in access to care, protect vulnerable communities, and create a more equitable future for all patients affected by allergic disease and immunodeficiency.
